# Causes of Increased Use of Closed Reduction and Internal Fixation for High-Energy-Related Traumatic Sacral Fractures

**DOI:** 10.1007/s00268-022-06876-4

**Published:** 2022-12-25

**Authors:** Yi-Hsun Yu, Ping-Jui Tsai, Chang-Heng Liu, I.-Jung Chen, Yung-Heng Hsu, Ying-Chao Chou, I.-Chuan Tseng

**Affiliations:** 1 Department of Orthopedic Surgery, Musculoskeletal Research Center, Chang Gung Memorial Hospital, Chang Gung University, Tao-Yuan, 33302 Taiwan; 2 Department of Orthopedic Surgery, Chang Gung Memorial Hospital, Taoyuan Branch, Taoyuan, Taiwan

## Abstract

**Background:**

Reasons for the increased use of closed reduction and internal fixation (CRIF) for traumatic sacral fractures (SFs) are unclear in the literature. Therefore, we aimed to report the annual changes in the number of patients, mechanisms of injury, fracture patterns, and fixation methods.

**Methods:**

In this retrospective study, we extracted data of 271 patients (mean age, 37.5 years) from the trauma register over an 8-year period. Annual records regarding the number of patients, injury mechanisms, fracture types, and treatment options were statistically analyzed to examine the interactions among these factors.

**Results:**

The number of patients with SFs increased significantly each year. The rate of admission to the intensive care unit after resuscitation was high (64.9%). Arbeitsgemeinschaft für Osteosynthesefragen (AO) type C pelvic ring injury (PRI), Dennis zone II injury, Roy-Camille type 2 injury, and U/H-type injury were the most common fracture types. Trans-iliac trans-sacral screws were mainly used in AO type B PRI, and their use significantly increased each year. For AO type C PRI, open reduction and internal fixation (ORIF) with rigid fixation was the main treatment, and the use of CRIF with iliosacral screws decreased each year. Stepwise statistical analysis revealed that the increase in AO type B PRI and ORIF for anterior PRI were the factors contributing to the increased use of CRIF for SFs.

**Conclusions:**

While the use of osteosynthesis for SFs is increasing, an increased use of CRIF for traumatic SFs has also been observed in clinical practice. This increase can be attributed to the increase in AO type B PRIs and ORIF for anterior PRIs.

## Introduction

Sacral fracture (SF), a common finding during trauma surveys in emergency departments, can result from low-energy injuries (leading to stress fractures), or high-energy trauma, such as falls from height or motor vehicle collisions. Recently, the diagnosis of SF has become more accurate and instantaneous because of the widespread use of multiplanar computed tomography (mCT) during trauma surveys [1, 2].

For high-energy-related SFs, treatment can be conservative or operative [1–4]. Osteosynthesis is recommended when the fracture pattern of the sacrum and pelvis reveals a deformity or an instability. Various reduction techniques (including closed reduction and internal fixation [CRIF] or open reduction and internal fixation [ORIF]) and implant options (including percutaneous screw osteosynthesis [PSO], plate osteosynthesis, triangular osteosynthesis [TO], and spinopelvic osteosynthesis [SPO]) have been used [5–10]. Future trends in osteosynthesis may shift more towards percutaneous, minimally invasive, and robotic-assisted procedures owing to advances in pelvic biomechanics, implants, and intraoperative imaging systems [11–13]. The recent increased use of CRIF as a definite treatment is one such trend [6]. However, the reasons for this change are unclear.

In this study, we reviewed the records of patients with high-energy-related traumatic SFs at a single medical institute over an 8-year period. The primary goal was to report the annual changes in the number of patients, mechanisms of injury, fracture patterns, and fixation methods. Additionally, the factors affecting the osteosynthesis type used for SFs were determined.

## Materials and methods

We hypothesized that SFs increased due to motor vehicle accidents, despite the fact that traffic safety has been repeatedly publicized throughout the past decade. Additionally, percutaneous osteosynthesis for high-energy related SFs increased during the same period. Between 2013 and 2021, consecutive patients with SFs were selected from the fracture registry database of our institute. The inclusion criteria were as follows: (1) fractures involving the sacrum, either an isolated SF or an SF accompanied by pelvic ring injury (PRI); (2) osteosynthesis for SF; (3) complete perioperative radiological assessments; and (4) complete follow-up until fracture union. Patients who received conservative treatment and had inadequate radiological assessments were excluded. The review of the medical records and radiological images was approved by our Institutional Review Board.

### Standardized resuscitation protocol

Since high-energy-related SFs are commonly accompanied by PRIs, the resuscitation protocol was similar to that of PRI. Once the patient was sent to our emergency department (ED), either directly from the trauma scene or via transfer from a primary medical institute, the resuscitation protocol was based on the Advanced Trauma Life Support guidelines. Patients with closed PRIs and shock received blood transfusion with packed red blood cells, fresh frozen plasma, and platelets in a 1:1:1 ratio. Furthermore, for those who were unresponsive to fluid and blood resuscitation, arterial embolization (AE) was performed first to stop retroperitoneal bleeding arising from the pelvic fracture.

In contrast, for those with open PRIs or SFs, surgical debridement, gauze packing of the open wound in the case of pelvic instability, and external fixation were performed following primary resuscitation. The blood transfusion protocol was similar to that for closed injuries. Life-saving surgical procedures, such as thoracotomy and laparotomy, were performed simultaneously or sequentially during damage-control orthopedic surgery. In the case of persistent hemodynamic instability, arterial embolization was performed after life-saving procedures to facilitate hemostasis in the pelvic region. The gauze pack was removed 24 h postoperatively; repeated debridement procedures were performed to reduce contamination. The timing for osteosynthesis was decided based on the patient’s hemodynamic status.

### Perioperative imaging

Preoperative imaging, including pelvic X-ray series (anteroposterior, inlet, and outlet views) and mCT, were mandatory for osteosynthesis planning. The position of the patient (supine or prone) during osteosynthesis, method of reduction (CRIF or ORIF), choice of implants, and the need for lumbar/sacral nerve root decompression were decided after the completion of image surveys. Postoperative pelvic X-ray series were obtained for each patient. Routine mCT for evaluation of reduction quality and implant positioning was performed after osteosynthesis as per protocol.

Additionally, preoperative bowel preparation was performed before PSO with iliosacral screw (ISS) or trans-iliac trans-sacral screw (TITS) placement for clearer real-time intraoperative fluoroscopic imaging.

### Applied classifications

PRIs were classified based on the Arbeitsgemeinschaft für Osteosynthesefragen (AO) classification system (2018 revision) into three types: type A, stable PRI; type B, partially unstable PRI; and type C, completely unstable PRI [13]. Three other classifications were also used to classify SFs: Dennis classification [14], Roy-Camille classification [6], and classification by morphology [4]. (Table 1).

**Table 1:** Applied classifications of high-energy-related sacral fractures

Classification		Description
Dennis [14]	Vertical fracture line of the sacrum in the coronal plane	Zone I: injuries located lateral to the foramina Zone II: injuries that involve the foramina Zone III: injuries medial to the foramina
Roy-Camille [15]	Transverse component of the sacrum in the sagittal plane according to its displacement	Type 1: a flexion-type injury with a resultant kyphotic deformity of the sacrum but without fracture displacement Type 2: a flexion-type injury, but with resultant posterior displacement of the cephalad segment relative to the caudad segment Type 3: an extension-type injury with resultant anterior translation of the cephalad segment
Morphology [4]	Combined transverse and vertical fractures of the sacrum in the coronal plane	H-type U type T type Lambda type

### Rehabilitation protocol

The rehabilitation protocol was individualized according to the fracture pattern, concomitant injuries, and preoperative functional status. Generally, toe-touch weight-bearing ambulation was allowed during the first 6 weeks after osteosynthesis for rotationally unstable SFs. Subsequently, progressive full weight-bearing ambulation was suggested. The aim of ambulation training was to enable assistance-free ambulation 12 months after osteosynthesis.

For patients with vertically unstable SFs, wheelchair-assisted ambulation was suggested for the first 6 weeks postoperatively if only limited fixation (i.e., PSO) was provided. However, if TO or SPO was performed, early weight bearing was allowed.

Drug prophylaxis for venous thromboembolization (VTE) was not routinely prescribed perioperatively. Mechanical prophylaxis with compressive socks was required for each patient when there were no contraindications, such as concomitant lower extremity injury, compromised lower extremity soft tissue, or vascular or nerve repair/graft procedures. Routine lower-extremity venous Doppler ultrasonography was performed for each patient 3 days after osteosynthesis to screen for VTE. mCT venography was performed if the screening was positive. Anticoagulants were prescribed if the VTE was confirmed.

### Statistical analysis

Statistical analyses were performed using SPSS software (version 21.0; SPSS Inc., Chicago, IL, USA). Continuous variables were compared using the independent t-test (two-tailed test); categorical variables were compared using the chi-squared test and Fisher’s exact test. Trend analysis was performed using the Pearson correlation test to determine the relationships between the selected parameters. Logistic regression analysis was used to assess the risk factors. Odds ratios (ORs) and the corresponding 95% confidence intervals (CIs) were used to analyze risks. Statistical significance was defined as *P *< 0.05.

## Results

**Fig. 1:**
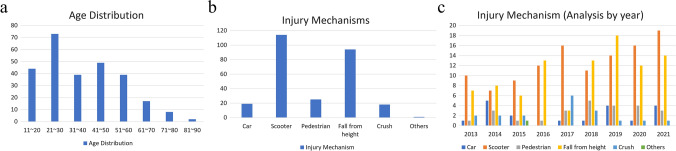
Demographic distribution of sacral fractures by **a** age, **b** injury mechanism, and **c** injury mechanism (by year)

**Fig. 2:**
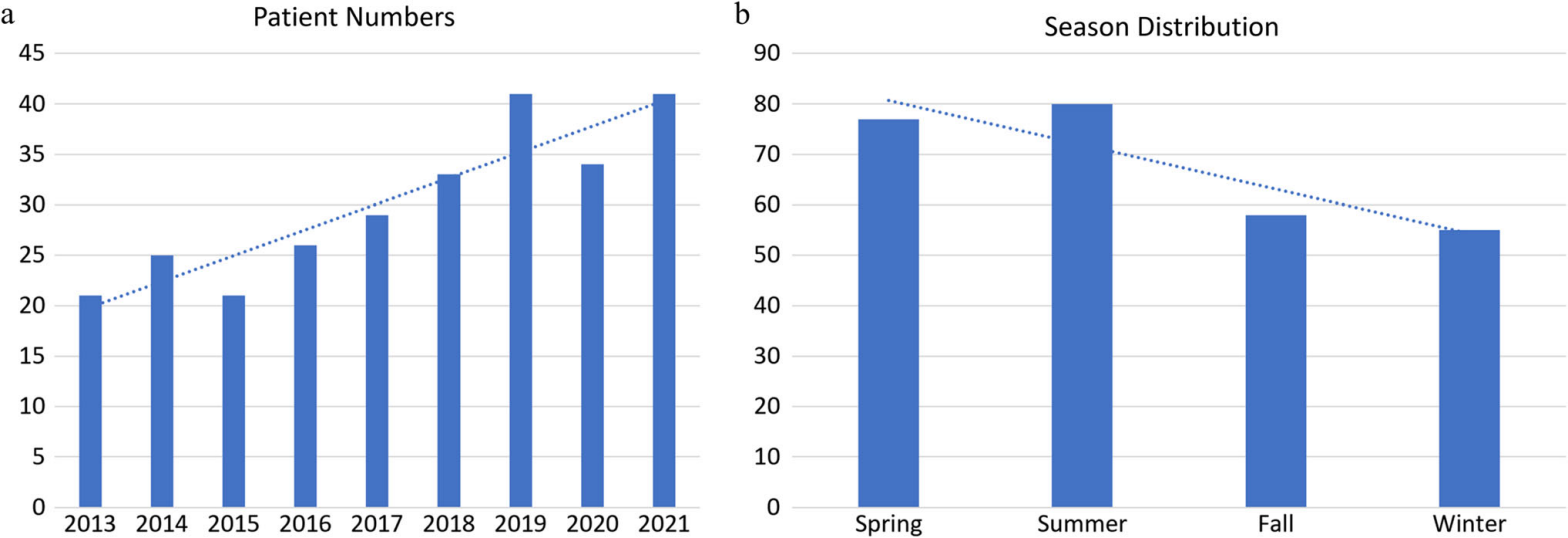
An analytical illustration of sacral fractures by **a** year and **b** season

During the study period, 271 patients sustained SFs from high-energy injuries. The demographic distribution of the enrolled patients is shown in Table 2. Most patients with traumatic SFs were between 21 and 30 years old, and there was a gradual decrease in the incidence of SF with age (*R*^2^ = 0.74, *P *= 0.006; Fig. 1a). Scooter accidents were the most common cause of injury each year and increased annually (*R*^2^ = 0.60, *P *= 0.004; Fig. 1b and c). Figure 2a shows a significant increase in the annual number of osteosynthesis procedures (*R*^2^ = 0.84, *P *< 0.001). The incidence of surgically treated SFs in the high-temperature seasons (spring and summer) was significantly higher than that in the low-temperature seasons (fall and winter) (*R*^2^ = 0.79, Fig. 2b).

**Table 2:** Demographic characteristics of patients with high-energy-related sacral fractures with applied osteosynthesis

Patient number	271
Sex (Male:Female)	140:131
Transferred from other hospitals	175
Age (years)	34.5 ± 16.2
*Injury scores*	
Injury severity score [median (IQR)]	20 (20)
New Injury Severity Score [median (IQR))	27 (20)
Revised Trauma Score	7.3 ± 1.1
*Concomitant injuries*	
Head	63 (23.2%)
Chest	122 (45.0%)
Abdomen	113 (41.7%)
Extremity fracture*	171 (63.1%)
Spine fracture (other than sacrum)	51 (18.8%)
Urogenital organs	38 (14.0%)
Rectum	10 (3.7%)
*Fracture classifications*	
AO (pelvic ring injury)	
Type A	1 (0.3%)
Type B	94 (34.7%)
Type C*	146 (64.9%)
*Dennis classification*	
Zone I	92 (33.9%)
Zone II*	166 (61.3%)
Zone III	12 (4.4%)
*Roy-Camille classification*	
1	21 (7.7%)
2*	39 (14.4%)
3	12 (4.4%)
*Morphology*	
U shape*	25 (9.2%)
H shape*	25(9.2%)
T shape	12 (4.4%)
Lambda shape	8 (3.0%)
Arterial embolization	104 (38.3%)
ICU admission	176 (64.9%)
ICU admission (days)	4.2 ± 6.1
Death	3 (1%)

Values are presented as mean ± SD or n (%)

^*^Statistical significance

*AO* Arbeitsgemeinschaft für Osteosynthesefragen, *ICU* Intensive care unit, *IQR* Interquartile range, *SD* Standard deviation

The rate of admission to the intensive care unit (ICU) after resuscitation was high (64.9%). Table 3 shows the conditions and concomitant injuries that warranted ICU admission. The rate of ICU admission was significantly higher for patients who presented with shock and underwent AE than for patients who presented with head trauma, chest trauma, or abdominal trauma (OR = 4.38, 95% CI = 1.23–15.56, *P *= 0.02). Moreover, among patients with head trauma, chest trauma, abdominal trauma, or a combination of these trauma types, those who underwent AE were significantly more likely to be admitted to ICU than were those who did not undergo AE (OR = 16.32, 95% CI = 4.83–55.10, *P *< 0.01). Among patients who presented with head trauma, abdominal trauma, or both and underwent AE, those with concomitant chest trauma were significantly more likely to be admitted to ICU than were those without concomitant chest trauma (OR = 4.77, 95% CI = 2.05–11.14, *P *< 0.01).

**Table 3:** Conditions and concomitant injuries that warranted intensive care unit admission of patients with traumatic sacral fractures

Conditions and injuries warranting ICU admission	Patient number	Percentage (%)
AE	22	12.5
HT	9	5
CT	15	8.5
AT	17	9.7
AE + HT	3	1.7
AE + CT	17	9.7
AE + AT	12	6.8
AE + HT + CT	8	4.5
AE + HT + AT	2	1.1
AE + CT + AT	25	14.2
AE + HT + CT + AT	15	8.5
HT + CT	7	4.0
HT + AT	1	0.6
HT + CT + AT	2	1.1
CT + AT	16	9.1
Others	5	2.8

*AE* Arterial embolization, *AT* Abdominal trauma (including hollow/solid organ injury and mesenteric artery injury), *CT* Chest trauma (including chest wall contusion and multiple rib fractures), *ICU* Intensive care unit, *HT* Head trauma (including subdural/subarachnoid/intracranial hemorrhage)

The most common fracture type according to each classification was AO type C PRI (OR = 3.45, CI = 1.93–6.12), Dennis zone II injury (zone I vs. zone III: OR = 12.4, *P *< 0.01; zone II vs. zone III, OR = 39.2, *P *< 0.001; predictive value [PV] = 74.7%), Roy-Camille type 2 injury (type 2 vs. type 3: OR = 4.3; PV = 68%), and U/H-type injury (U/T vs. T: OR = 2.75, *p *< 0.05, PV = 75%). Among all AO type B PRIs, type B 2.1 was the most common (OR = 35.3, *P *< 0.001, PV = 86%), and among all AO type C PRIs, type C 1.3 was the most common fracture type (OR = 1.93, *P *< 0.05, PV = 83.7%).

**Fig. 3:**
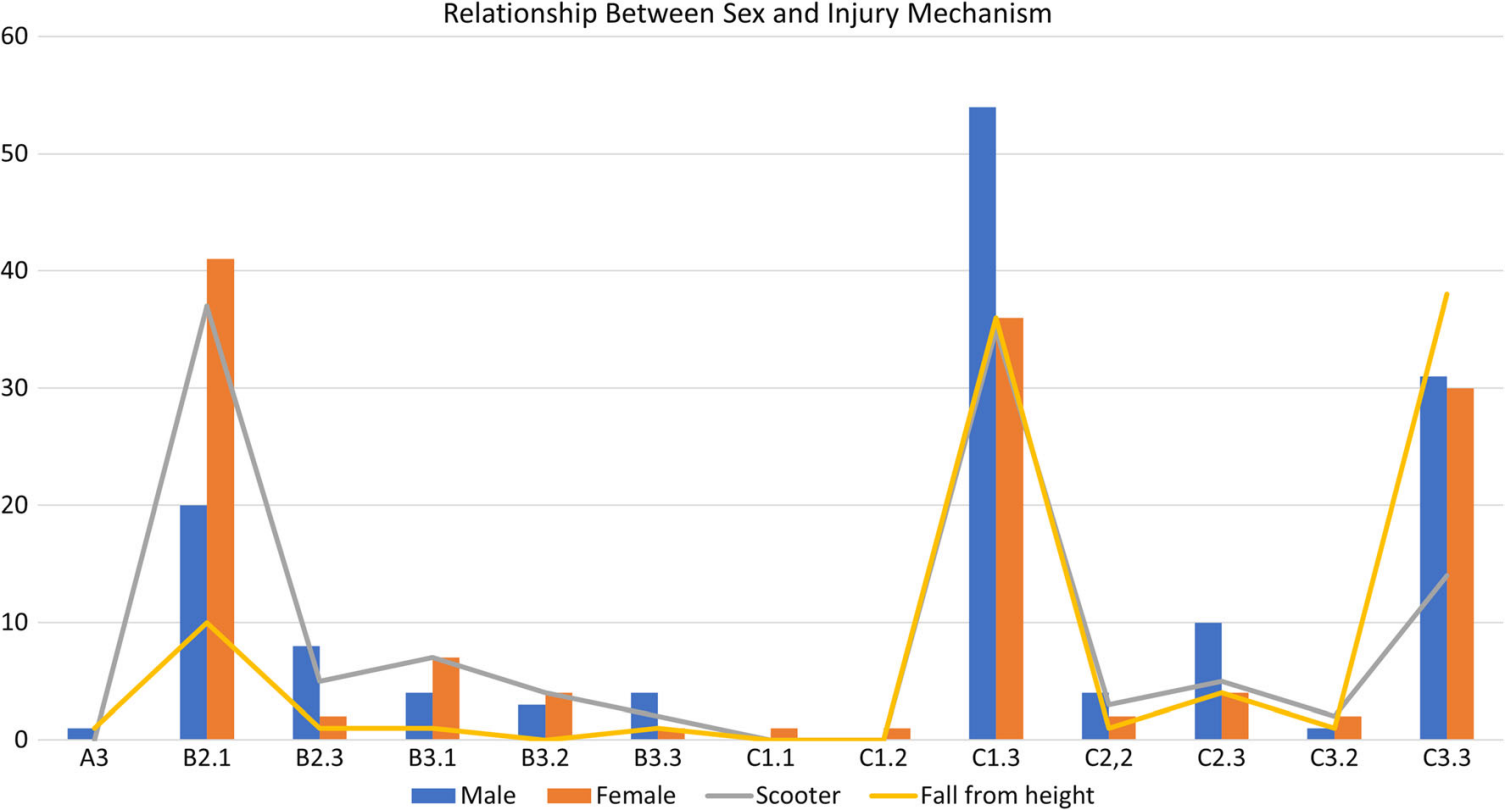
Schematic distribution of sex, injury mechanism, and fracture classification (Arbeitsgemeinschaft für Osteosynthesefragen classification for pelvic ring injury)

The relationships between sex, mechanism of injury (scooter accident and fall from height) and fracture type (AO classification) were examined (Fig. 3). There was no significant difference between the sexes and mechanisms of injury. Women had a higher rate of AO type B2.1 PRI (OR = 2.12, *P *< 0.05). Scooter accidents were prone to cause AO type B2.1, B2.3, and C1.3 PRIs (all *P *< 0.05, PV = 67.3%).

### Osteosynthesis-related analysis

**Fig. 4:**
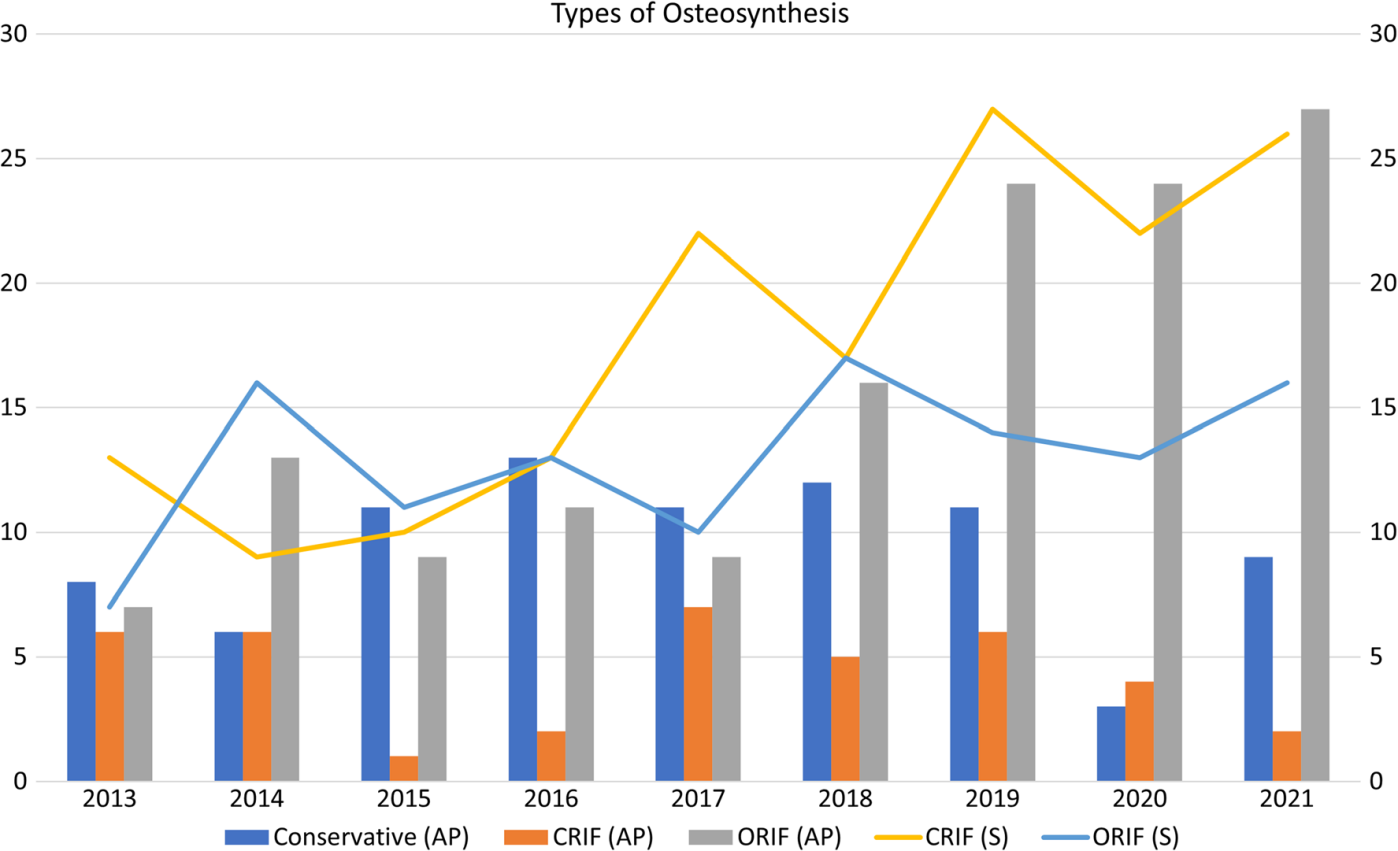
Annual analysis of fixation methods for anterior pelvic ring (AP) and sacrum (S). *CRIF* Closed reduction and internal fixation, *ORIF* Open reduction and internal fixation

Regarding the choice of fixation implants (Fig. 4), PSO using TITS was primarily applied in AO type B PRIs (*P *< 0.05) and its use significantly increased each year (*R*^2^ = 0.85, *P *= 0.01). For AO type C PRIs, TO, sacral plate (SP) and SPO were the main fixation implants (*P *< 0.05), and the trend of PSO by placing ISS decreased each year (*R*^2^ = 0.60, *P *= 0.02).

**Fig. 5:**
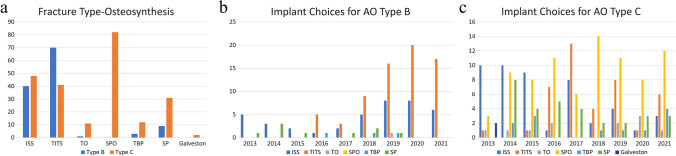
**a** Summary of applied methods of osteosynthesis for sacral fractures. Applied methods of osteosynthesis for **b** AO type B and **c** C pelvic ring injuries. *AO* Arbeitsgemeinschaft für Osteosynthesefragen, *ISS* Iliosacral screw, *TITS* Trans-iliac trans-sacral screw, *TO* Triangular osteosynthesis, *SPO* Spinopelvic osteosynthesis, *TBP* Tension band plate, *SP* Sacral plate

The distribution of osteosynthesis methods for sacral and anterior PRIs is shown in Fig. 5. CRIF for SFs increased each year (*R*^2^ = 0.68, *P *= 0.03). A similar increasing trend was observed in ORIF for anterior PRI (*R*^2^ = 0.71, *P *= 0.001). The factors related to this finding were further analyzed in a stepwise manner. In the first step, descriptive statistical analysis was performed for the chosen factors (fracture classification, Morel-Lavallée lesion, closed/open fracture, sex, and CRIF/ORIF for anterior PRI). Fracture classifications (AO, Dennis, and Roy-Camille) and CRIF/ORIF for anterior PRIs were determined to be significant by the chi-squared test, Fisher exact test, and independent t-test; this was confirmed by the Pearson correlation test in the second step. Lastly, in the logistic regression test, AO type B PRI and ORIF for anterior PRI were identified as factors that caused the increased use of CRIF for SFs (*R*^2^ = 0.482).

## Discussion

There has been a recent increase in the incidence of SFs related to high- or low-energy trauma [16, 17]. High-energy-related traumatic SFs are often accompanied by other major injuries, such as thoracic cavity injuries, extremity fractures, and PRIs [2, 18, 19]. Additionally, these fractures may cause considerable morbidity and mortality, even after appropriate treatment [2, 18]. Therefore, ICU admission is often required. The results of our 8-year, single-center study indicated an annual increase in the number of high-energy-related traumatic SFs. Furthermore, the degree of fracture instability and the need for osteosynthesis also increased. However, despite the increasing degree of instability, the approach to SFs tended to be more percutaneous in nature. We found that this trend may be related to the increase in AO type B PRIs and increased use of ORIF for anterior PRIs.

Scooters are the main mode of transport in Taiwan (an estimated 97.7 motorcycles per every 100 persons) [20]. Traffic collisions with scooters may result in high morbidity and mortality. A report from the Ministry of Transportation and Comminutions demonstrated that 376,697 scooterists were fatally injured in 2014, accounting for 51% of total traffic fatalities [20]. Scooter collisions were also a leading cause of high-energy-related traumatic SFs that underwent osteosynthesis in our study, the incidence of which increased annually. Additionally, AO type B2.1 and C1.3 PRIs were the most common fracture patterns found among scooterists. For AO type B PRI, the strategy of osteosynthesis is to correct the internally rotated deformity of the affected hemipelvis through an anterior approach. Subsequently, PSO with TITS(s) was applied to the SF for posterior pelvic ring fixation. We used a similar surgical approach for complete unilateral longitudinal SF as in AO type C1.3 PRI. Surgical procedures were mostly performed under skeletal traction, which provided a steady axial force to counteract cranial-caudal discrepancy. Therefore, we only had to focus on correcting rotational deformities during ORIF of SFs with anterior PRIs and CRIF of SFs with posterior PRIs. Hence, as the number of SFs due to scooter collisions increased, the use of CRIF with TITS also increased.

Minimally invasive osteosynthesis is a current trend in the field of orthopedic traumatology. PSO by insertion of ISSs is one of the minimally invasive techniques for fixation of SFs with posterior PRI. However, the disadvantages of placing ISS for SFs include short screw usage, inadequate fixation strength, and the fact that the trajectory of the screw placement is out of line with the plumb line of the fracture [21, 22]. In contrast, TITS placement is an alternative method of PSO that is applied from one ilium to the other and involves the fixation of six cortices (four iliac and two sacral cortices). In combination with anterior pelvic ring fixation, the placement of a long screw perpendicular to the longitudinal SF line using a minimally invasive technique provides adequate fixation stiffness for AO type B PRI and unilateral SFs. This approach also avoids soft tissue complications [23, 24]. In our study, the application of TITS for SFs increased, especially for rotationally unstable PRIs, and had a positive relationship with the increased rate of ORIF for anterior PRIs. We believe that after secure fixation of the anterior pelvic ring, SF could be treated with one to two TITS(s) to achieve a stable pelvic ring and sacrum. Additionally, the minimally invasive approach to SFs has the advantage of less soft tissue compromise and surgical site infection [4, 15].

Despite the increasing use of minimally invasive procedures for SFs, the application of ORIF to connect the lumbar spine to the unstable pelvic ring (i.e. TO and SPO) has remained a major osteosynthesis procedure for vertically unstable SFs. Fall from height is a leading cause of high-energy traumatic SF [25] and was the second commonest etiology in our cohort. Additionally, we observed a trend towards a higher rate of surgical treated SFs in the high-temperature seasons (spring and summer) compared to the low-temperature seasons (fall and winter). We postulated that this finding might be associated with a higher incidence of intentional falls. Intentional falling (suicide by jumping) is considered a major cause among falls in high-energy traumatic SFs. [25] Although some studies indicates that the seasonal change has no correlation with the incidence of suicide by jumping [26, 27], some reports support this finding [28, 29]. Although it is unclear why seasonal change causes a higher rate of suicide by jumping, the temperature conversion, environmental change, and serotonin shift are possible causes [28–32].

This study has some limitations, despite efforts to avoid them. Our use of a retrospective study design may have resulted in data-record bias. Additionally, the choice of fixation method mainly depended on the surgeon’s preferences, which might have led to selection bias. However, five orthopedic surgeons were involved in the operations, and one senior surgeon performed most of the surgical procedures (81.9%). Finally, functional recovery is closely related to the fracture pattern, quality of fracture reduction, and selected treatment. The radiological and functional outcomes after the treatments were not fully reported in this study. Future studies should focus on the surgical outcomes of each fracture type and the corresponding treatment.

In conclusion, the number of high-energy-related traumatic SFs has increased over the past 8 years, and early ICU admission might be necessary because of concomitant injuries, particularly for patients who have sustained chest trauma and those who have undergone AE. A high degree of instability or deformity was the most common feature according to each classification. Osteosynthesis for SFs tends to be a percutaneous method and seems correlated with increasing AO type B PRIs and ORIF for anterior PRIs. While PSO with TITS has been increasingly applied in SFs with AO type B PRIs, the placement of ISS for AO type C PRIs has decreased.
